# Impact of Vaping on Hospital Outcomes in Patients With Acute Respiratory Distress Syndrome

**DOI:** 10.7759/cureus.89138

**Published:** 2025-07-31

**Authors:** Danny V Tran, Nicholas W Sheets, Jocelyn Limas

**Affiliations:** 1 Internal Medicine, HCA Florida Trinity Hospital, Trinity, USA; 2 Trauma and Acute Care Surgery, Riverside Community Hospital, Riverside, USA; 3 Graduate Medical Education, Riverside Community Hospital, Riverside, USA

**Keywords:** acute respiratory distress syndrome [ards], e-cigarette, e-cigarette and vaping product use associated lung injury (evali), vape, vaping-associated lung injury

## Abstract

Objective

Introduced in the early 2000s, e-cigarettes and vaping have risen in popularity, leading to a notable increase in acute lung injury and the coining of electronic cigarette or vaping-associated lung injury (EVALI). Current studies have examined outcomes in EVALI specifically; however, data on the impact of vaping in acute respiratory distress syndrome (ARDS) is lacking. Thus, the objective of this study is to investigate the impact of vaping on hospital outcomes in patients with ARDS.

Materials and methods

This study is a retrospective chart review utilizing the Hospital Corporation of America (HCA) Healthcare Far West Division database. Unique patient encounters between January 1, 2016, and July 31, 2022, were included if they were associated with a diagnosis of acute respiratory distress syndrome (ARDS) and had documented vaping status. Pediatric patients under the age of 18 years were excluded. Due to the retrospective nature of the study, no interventions were performed. Outcomes were stratified and compared based on vaping status. Demographics and comorbidities were controlled for in the analysis.

Measurements

Patient demographics and medical comorbidities were extracted and analyzed. Key hospital outcomes of interest include use of non-invasive positive pressure ventilation (NIPPV), use of mechanical ventilation, hospital and intensive care unit (ICU) length of stay (LOS), and mortality. Statistical analysis of comorbidities was performed using chi-square analyses and Fisher’s exact test between the two groups. Multinomial logistic regression was used to analyze the type of respiratory support required, while negative binomial regression was used to analyze hospital and ICU length of stay. Binary logistical regression with Firth correction was used to analyze differences in mortality. Statistical significance for all analyses was defined as a p-value of less than 0.05. Odds ratios were calculated with a 95% confidence interval. All analyses controlled for age, BMI, sex, medical comorbidities, and smoking status.

Results

A total of 269 patients were identified, of whom 42 vaped. Statistical analysis revealed no significant differences in demographics between those who vaped and those who did not. Fifty-seven patients required NIPPV, of whom 12 vaped. Eighty patients required mechanical ventilation, of whom seven vaped. Binary logistic regression with a Firth correction revealed a statistically significant decrease in odds (odds ratio {OR}: 0.377; 95% CI: 0.145, 0.979; p=0.045) of requiring mechanical ventilation in patients who vaped compared to those who did not. There were 15 mortalities, of whom four vaped. Binary logistic regression did not reveal statistical significance in mortality. The average hospital length of stay was 8.94 days, and the average length of stay (ICU LOS) was 5.48 days. Negative binomial regression revealed no statistically significant difference in both hospital and ICU LOS between patients who vaped and those who did not.

Conclusion

While statistical analysis did reveal a significantly decreased odds ratio of requiring mechanical ventilation in patients who vaped, the power of this and other findings was low, given a sample size of 42 who vaped. Despite its limitations, the study shares some preliminary insight into vaping and ARDS for future studies.

## Introduction

Since its introduction in the early 2000s, the electronic cigarette or e-cigarette has rapidly gained popularity, particularly among adolescents [1,2]. E-cigarettes are typically marketed as an alternative to cigarette smoking or a stepping stone to smoking cessation with lower nicotine content; however, there is concern that they are leading non-smokers to smoke combustible cigarettes [3]. These devices aerosolize the solution into vapor that is inhaled. These solutions can contain a mixture of nicotine, tetrahydrocannabinol (THC), flavoring, and additives; however, the exact quantities and ratios are not regulated and can differ across brands and products [4]. In fact, some studies have shown that e-cigarettes can have higher aerosolized nicotine content compared to traditional combustible cigarettes [4-6]. The resultant composition of the aerosol inhaled is highly variable and likely due to differing device specifications between brands and even models within those brands, as well as in contents and concentrations of the solutions used [4].

Vitamin E acetate has been found at the primary site of injury in the lungs of patients who used e-cigarettes and has been identified as a possible hazardous material [7]. As an additive, vitamin E acetate is a very sticky substance that stays in the lungs, causing injury [7]. Currently, the Centers for Disease Control and Prevention (CDC) recommends avoiding products containing vitamin E acetate, with some states banning those products altogether [8].

The exact pathophysiologic effects of e-cigarette aerosol are actively being investigated, with some reports suspecting similar downregulation of the chromosomal fragile site gene, WW domain-containing oxidoreductase (WWOX), as seen in cigarette smokers [9]. WWOX downregulation has been associated with increased protein leakage, neutrophil influx, and increased inflammatory cytokine production and thus exacerbating the inflammatory process and lung injury [9].

The rise in e-cigarette use has led to an increased incidence of acute lung injury, leading to electronic cigarette or vaping-associated lung injury (EVALI). As of 2020, 2,807 cases had been reported to the CDC, and some studies have published their findings and outcomes [8]. One study by Hayes Jr et al. reported 2,708 confirmed cases of EVALI as of January 21, 2020, of which 1,604 (59.2%) required admission to the intensive care unit (ICU) [10]. Many of those patients (88.5%) required respiratory support, with some undergoing intubation (36.1%) and extracorporeal membrane oxygenation (6.7%) [10].

Werner et al. also utilized CDC data to examine the population demographics of the 2,558 patients, particularly the fatal and non-fatal subgroups [11]. Results indicated that fatal and non-fatal cases were predominantly male (53% and 67%, respectively), non-Hispanic white (80% and 61%, respectively), had a history of asthma (23% and 8%, respectively), cardiac disease (65% and 41%, respectively), and a mental health condition (65% and 41%, respectively) [11]. Fatal cases were predominantly older than 35 years (73%), while non-fatal cases had a lower proportion of those above 35 years old (22%) [11].

Studies thus far have identified patient demographics and hospital outcomes of those diagnosed with EVALI, particularly in fatal versus non-fatal cases; however, data on acute respiratory distress syndrome (ARDS) and vaping are lacking. We suspect that vaping may have either a negative or no impact on hospital outcomes in patients with ARDS. This study aimed to compare the factors between patients who vaped and did not vape to further examine the impact of vaping on ARDS outcomes.

## Materials and methods

Data collection

We conducted a retrospective chart review to identify hospitalized patients with ARDS with documented vaping status between January 1, 2016, and July 31, 2022. The data were obtained from the Hospital Corporation of America (HCA) Healthcare Far West Division database. This study was determined to be IRB-exempt and in compliance with the code of ethics when reviewed by HCA Healthcare’s Centralized Algorithms for Research Rules on IRB Exemption Committee. Patient consent was not required for this study, given its retrospective design and the omission of all personal or identifying information.

Patient selection and outcome measures

Patients with a diagnosis of ARDS, as defined by the International Classification of Diseases, Tenth Revision (ICD-10) codes J80, J80.0, J80.01, J80.02, or J80.03, and documented vaping status, were included in the study. Vaping status was obtained by review of all clinical notes by the data extraction team. Patients with a documented status of "unknown" or those without a documented vaping status were excluded from the study. Patients below 18 years of age were also excluded from the study. Patient demographics (age, sex, and body mass index), medical comorbidities, smoking history, type of respiratory support required during hospitalization, and hospital and ICU outcomes were extracted, reviewed, and analyzed. Comorbidities included in this study were chronic obstructive pulmonary disease (COPD), bronchitis, emphysema, asthma, interstitial lung disease, pulmonary fibrosis, sepsis, coronary artery disease (CAD), congestive heart failure (CHF), pulmonary hypertension, and chest trauma. Respiratory support was defined as the use of either non-invasive positive pressure ventilation (NIPPV) or mechanical ventilation. The study population was divided by vaping status as follows: patients who vaped versus patients who did not vape. Hospital outcomes included length of stay, ICU admission, and overall mortality. ICU outcomes included unit length of stay and unit mortality.

Statistical analyses

Statistical analysis of demographics and comorbidities was done using chi-square analyses and Fisher’s exact test. The analysis used was dependent on sample size. Those with a sufficient sample size underwent chi-square analysis, while the others underwent Fisher’s exact test. Significant differences in age and BMI between the two groups were analyzed using two independent sample t-tests. Binary logistical regression with Firth correction was used to assess for significant differences in mortality between the two groups. Multinomial logistic regression was used to analyze the type of respiratory support required, while negative binomial regression was used to analyze hospital and ICU length of stay. Ordinal logistic regression was used to analyze the order of ventilation duration defined as less than 24 hours, 24-96 hours, and greater than 96 hours. Statistical significance for all analyses was defined as a p-value of less than 0.05. Odds ratios were calculated with a 95% confidence interval. All analyses controlled for age, BMI, sex, medical comorbidities, and smoking status.

## Results

We initially identified 285 encounters with the ARDS diagnosis and documented vaping status. A total of 14 encounters were removed due to missing demographic information, and two encounters were removed as they involved a patient with another encounter in the pool. A final total of 269 unique patient encounters were included in the analysis.

There were 227 patients (84.39%) who did not vape and 42 patients (15.61%) who vaped. Statistical analyses, including independent sample t-tests, chi-square analyses, and Fisher’s exact tests, where appropriate, revealed no significant differences in demographics and comorbidities between patients who vaped and did not vape (Table 1).

**Table 1:** Demographics and medical comorbidities. *Chi-square tests were conducted to compare vaper with non-vaper on binary outcomes with sufficient sample size in each cell. **Fisher’s Exact tests were conducted to compare vaper with non-vaper on binary outcomes where some cells don’t have sufficient sample size (i.e., n<5). ***Two independent sample t tests were conducted to compare vaper with non-vaper on numeric variables. Statistical significance defined as p<0.05. COPD: chronic obstructive pulmonary disease; CAD: coronary artery disease; CHF: congestive heart failure

Variables	Category	Vape				p-Value
		No (n=227)		Yes (n=42)		
		n	%	n	%	
Sex*	Female	84	37.00	18	42.86	0.4727
	Male	143	63.00	24	57.14	
Comorbidity*	No	44	19.38	9	21.43	0.7595
	Yes	183	80.62	33	78.57	
COPD*	No	163	71.81	29	69.05	0.7164
	Yes	64	28.19	13	30.95	
Bronchitis**	No	225	99.12	42	100.00	1.0000
	Yes	2	0.88	0	0.00	
Emphysema**	No	225	99.12	42	100.00	1.0000
	Yes	2	0.88	0	0.00	
Asthma*	No	188	82.82	32	76.19	0.3066
	Yes	39	17.18	10	23.81	
Interstitial lung disease**	No	226	99.56	42	100.00	1.0000
	Yes	1	0.44	0	0.00	
Pulmonary fibrosis**	No	226	99.56	41	97.62	0.2884
	Yes	1	0.44	1	2.38	
Sepsis*	No	154	67.84	31	73.81	0.4433
	Yes	73	32.16	11	26.19	
CAD*	No	172	75.77	35	83.33	0.2851
	Yes	55	24.23	7	16.67	
CHF*	No	143	63.00	32	76.19	0.0994
	Yes	84	37.00	10	23.81	
Pulmonary hypertension*	No	204	89.87	35	83.33	0.2165
	Yes	23	10.13	7	16.67	
Chest trauma**	No	226	99.56	42	100.00	1.0000
	Yes	1	0.44	0	0.00	
Variables		Mean	SD	Mean	SD	p-Value
Age*** (years)		52.02	15.57	50.79	17.86	0.6459
Metric BMI***		31.07	11.16	29.60	9.72	0.4219

There were 15 reported mortalities in this study, of whom four vape (Table 2). There was no significant association between vaping status and mortality when analyzed using binary logistic regression with a Firth correction (χ^2^{1}=1.904, p=0.1676, OR=2.198, 95% CI: 0.718, 6.727) (Table 3). There was also no significant association of control variables with mortality (Table 3). It is important to note that due to the power with a sample size of only four, this finding is questionable.

**Table 2:** Hospital outcomes including length of stay, ICU admission, and overall mortality.

Variables	Category	Total (n=269)		Vape			
				No (n=227)		Yes (n=42)	
		n	%	n	%	n	%
ICU admission	No	144	53.53	119	52.42	25	59.52
	Yes	125	46.47	108	47.58	17	40.48
Mortality	No	254	94.42	216	95.15	38	90.48
	Yes	15	5.58	11	4.85	4	9.52
Respiratory support	Mechanical	80	29.74	73	32.16	7	1.67
	Non-invasive	43	15.99	34	14.98	9	2.14
	None	146	54.28	120	52.86	26	61.90
Variables		Mean	SD	Mean	SD	Mean	SD
Hospital length of stay (days)		8.94	12.27	9.29	12.73	7.10	9.38
ICU length of stay (days)		5.48	6.85	5.70	7.11	4.06	4.83

**Table 3:** Mortality (binary logistic regression with Firth correction). RS: respiratory support: DF: degrees of freedom; Pr: p-value; ChiSq: chi-square Statistical significance is defined as p<0.05.

Analysis of penalized maximum likelihood estimates						Odds ratio estimates		
Parameter	DF	Estimate	Standard error	Wald chi-square	Pr > ChiSq	Point estimate	95% Wald confidence limits	
Intercept	1	-1.8385	1.3542	1.8431	0.1746	-	-	-
Vape	1	0.7876	0.5707	1.904	0.1676	2.198	0.718	6.727
Age	1	-0.00856	0.0152	0.3157	0.5742	0.991	0.962	1.022
BMI	1	-0.0123	0.0264	0.2155	0.6425	0.988	0.938	1.04
Sex (male)	1	0.4309	0.5462	0.6224	0.4301	1.539	0.528	4.488
Comorbidity	1	0.0919	0.6281	0.0214	0.8837	1.096	0.32	3.754
Smoking	1	-0.7591	0.5127	2.1919	0.1387	0.468	0.171	1.279

A total of 123 patients required ventilator support from either mechanical or NIPPV (Table 2). Eighty patients required mechanical ventilation, of which seven vape. Forty-three patients required NIPPV, of which nine vape. Analysis with multinomial logistic regression indicated that patients who vaped had significantly decreased odds (χ^2^{1}=4.017, p=0.045; OR=0.377, 95% CI: 0.145, 0.979) of requiring mechanical ventilation (Table 4). Similar analysis of NIPPV use revealed no significant association (χ^2 ^{1}=0.438, p=0.508). Similar to above, the power of these findings is low and thus questionable given the small sample sizes of seven and nine.

**Table 4:** Respiratory support (multinomial logistic regression). RS: respiratory support; DF: degrees of freedom; Pr: p-value; ChiSq: chi-square Statistical significance is defined as p<0.05.

Analysis of maximum likelihood estimates							Odds ratio estimates		
Parameters	RS type	DF	Estimate	Standard error	Wald chi-square	Pr > ChiSq	Point estimate	95% Wald confidence limits	
Intercept	Mechanical	1	1.2359	0.8359	2.1863	0.1392	-	-	-
	Non-invasive	1	-4.7829	1.2546	14.5332	0.0001	-	-	-
Vape	Mechanical	1	-0.9756	0.4868	4.0168	0.0451	0.377	0.1450	0.9790
	Non-invasive	1	0.2972	0.4493	0.4377	0.5083	1.346	0.5580	3.2470
Age	Mechanical	1	-0.00798	0.00966	0.6818	0.4090	0.992	0.9730	1.0110
	Non-invasive	1	0.0187	0.013	2.0508	0.1521	1.019	0.9930	1.0450
BMI	Mechanical	1	0.00521	0.0153	0.1157	0.7338	1.005	0.9750	1.0360
	Non-invasive	1	0.0427	0.0148	8.3787	0.0038	1.044	1.0140	1.0740
Sex	Mechanical	1	0.2781	0.3156	0.7767	0.3781	1.321	0.7110	2.4510
	Non-invasive	1	-0.0209	0.366	0.0033	0.9545	0.979	0.4780	2.0070
Comorbidity	Mechanical	1	-1.3386	0.3566	14.0933	0.0002	0.262	0.1300	0.5270
	Non-invasive	1	0.9284	0.7795	1.4186	0.2336	2.53	0.5490	11.6600
Smoking	Mechanical	1	-0.9071	0.324	7.8392	0.0051	0.404	0.2140	0.7620
	Non-invasive	1	0.345	0.4762	0.5249	0.4688	1.412	0.5550	3.5910

Medical comorbidities collectively were a significant predictor for mechanical ventilation (χ^2^ {1}=14.093, p=0.0002) with a decreased odds ratio of 0.262 (95% CI: 0.130, 0.527). Similarly, smoking was a significant predictor for mechanical ventilation​​​​​ (χ^2^ {1}=7.839, p=0.005) with a decreased odds ratio of 0.404 (95% CI: 0.214, 0.762). Body mass index (BMI) was identified as a significant predictor of non-invasive positive pressure ventilator (NIPPV) use (χ^2^​​​​​​​ {1}=8.379, p=0.0038) with a slightly increased odds ratio of 1.044 (95% CI: 1.014, 1.074) times per unit increase of BMI (Table 4). All other control variables were not significantly associated with either mechanical ventilation or NIPPV (Table 4).

The overall mean hospital length of stay was 8.94 days. The mean hospital length of stay for patients who vaped was 7.10 days, and 9.29 days for patients who did not. The overall mean ICU length of stay was 5.48 days. Mean ICU length of stay in patients who vaped was 4.06 days compared to 5.70 days in those who did not (Table 2). Negative binomial regression showed no statistically significant difference in hospital (χ^2 ^{1}=1.900, p=0.169) and ICU (χ^2^ {1}=0.500, p=0.479) length of stay between those who vaped and those who did not. Control variables were also not significantly associated with hospital and ICU length of stay (Table 5).

**Table 5:** Hospital and ICU length of stay (negative binomial regression). LOS: length of stay; RS: respiratory support; DF: degrees of Freedom; Pr: p-value; ChiSq: chi-square Statistical significance defined as p<0.05.

Analysis of penalized maximum likelihood estimates								Contrast estimate results			
Parameter	DF	Estimate	Standard error	95% Wald confidence limits		Wald chi-square	Pr > ChiSq	Estimate	Standard error	95% Wald confidence limits	
Hospital LOS											
Intercept	1	2.1513	0.3328	1.4991	2.8035	41.8	<0.0001	-	-	-	-
Vape	1	-0.2413	0.1752	-0.5847	0.1021	1.9	0.1685	0.7856	0.1377	0.5573	1.1075
Age	1	0.0053	0.0039	-0.0024	0.0131	1.85	0.1743	1.0054	0.0040	0.9976	1.0131
BMI	1	-0.0077	0.0054	-0.0182	0.0029	2.02	0.1549	0.9924	0.0053	0.9819	1.0029
Sex	1	-0.0455	0.1278	-0.296	0.2049	0.13	0.7215	0.9555	0.1221	0.7438	1.2274
Comorbidity	1	0.2237	0.16	-0.0898	0.5372	1.96	0.1619	1.2507	0.2001	0.9141	1.7113
Smoking	1	-0.1864	0.1409	-0.4625	0.0898	1.75	0.186	0.8300	0.1169	0.6297	1.0940
Dispersion	1	0.8985	0.0816	0.752	1.0735	-	-	-	-	-	-
ICU LOS											
Intercept	1	1.2467	0.5176	0.2321	2.2613	5.8	0.016	-	-	-	-
Vape	1	-0.2156	0.3044	-0.8123	0.381	0.5	0.4787	0.8060	0.2454	0.4439	1.4637
Age	1	0.0054	0.0063	-0.007	0.0177	0.72	0.3955	1.0054	0.0063	0.9930	1.0179
BMI	1	0.0079	0.0096	-0.0109	0.0267	0.69	0.4075	1.0080	0.0097	0.9892	1.0271
Sex	1	-0.1677	0.2017	-0.5629	0.2276	0.69	0.4058	0.8456	0.1705	0.5695	1.2556
Comorbidity	1	0.2412	0.2263	-0.2023	0.6847	1.14	0.2865	1.2728	0.2880	0.8168	1.9833
Smoking	1	-0.1574	0.209	-0.567	0.2522	0.57	0.4513	0.8543	0.1786	0.5672	1.2868
Dispersion	1	0.8962	0.1327	0.6705	1.1979	-	-	-	-	-	-

Of the 80 patients who required mechanical ventilation, 23 patients required it for less than 24 hours, 25 patients required it for 24-96 hours, and 22 patients required it for greater than 96 hours. Of those who vaped, three patients required mechanical ventilation for less than 24 hours, two patients required it for 24-96 hours, and two patients required it for greater than 96 hours (Table 2). Ordinal logistic regression revealed no significant association of vaping with length of mechanical ventilation (χ^2^​​​​​​​ {1}=2.494, p=0.114). It is important to note that due to the sample size and power, the validity of this finding is questionable. When controlling for covariates, both comorbidity (χ^2^​​​​​​​ {1}=11.981, p=0.0005) and smoking (χ^2^​​​​​​​ {1}=12.709, p=0.0004) were found to have a significant association with the likelihood of the order of ventilation days. Both comorbidity (OR=3.138, 95% CI: 1.642, 5.996) and smoking (OR=2.964, 95%CI: 1.631, 5.386) had significantly increased odds of requiring higher-order ventilation days in patients who reported vaping compared to those who did not (Table 6).

**Table 6:** Ventilation duration order (ordinal logistic regression). RS: respiratory support; DF: degrees of freedom; Pr: p-value; ChiSq: chi-square Statistical significance is defined as p<0.05.

Analysis of penalized maximum likelihood estimates						Odds ratio estimates		
Parameter	DF	Estimate	Standard error	Wald chi-square	Pr > ChiSq	Point estimate	95% Wald confidence limits	
Intercept	1	-1.024	0.779	1.7278	0.1887	-	-	-
Intercept	1	-0.3766	0.7769	0.2349	0.6279	-	-	-
Intercept	1	0.493	0.7851	0.3944	0.5300	-	-	-
Vape	1	0.736	0.466	2.4943	0.1143	2.0880	0.8370	5.2040
Age	1	0.0116	0.00924	1.5829	0.2083	1.0120	0.9940	1.0300
Metric BMI	1	-0.00059	0.0135	0.0019	0.9652	0.9990	0.9730	1.0260
Sex (male)	1	-0.2878	0.3088	0.8683	0.3514	0.7500	0.4090	1.3740
Comorbidity	1	1.1436	0.3304	11.981	0.0005	3.1380	1.6420	5.9960
Smoking	1	1.0865	0.3048	12.7085	0.0004	2.9640	1.6310	5.3860

## Discussion

The findings from this study suggest that vaping is not associated with a difference in NIPPV use, mortality, hospital and ICU length of stay, and duration of mechanical ventilation in patients hospitalized with ARDS. However, it is important to note that the sample size of patients who vape is very small, likely impacting results. Most surprising is the odds ratio in patients who vaped, requiring mechanical ventilation. We initially hypothesized that vaping would have either a similar or increased odds ratio regarding mechanical ventilation, not decreased as seen in this study. We attributed this unexpected finding to the small sample size. The confidence interval and standard error of this finding were very high and large, considering that only seven of the 42 patients who reported vaping required mechanical ventilation. This effect is also seen in the statistical analysis of control variables. Other unexpected findings include those related to medical comorbidities and smoking, which were significantly associated with decreased odds of requiring mechanical ventilation. These findings can again be attributed to the small sample size, which likely skewed the data, resulting in statistical significance that is opposite of what is expected.

We suspect that the inadequate sample size may stem from the lack of reporting of vaping status. A study by Rubenfeld et al. reported the incidence of ARDS of 86 per 100,000 person-years, or approximately 190,000 cases per year in the United States as of 2005 [12]. Despite using the HCA Healthcare Far West Division database and a six-year date range, only 285 patients with ARDS and documented vaping status were obtained for this study, of which only 42 vaped. This discrepancy between the incidence of ARDS per year and our sample size over six years suggests that recording of vaping status may not be routine or consistent, likely due to the novel popularity of vaping and lack of evidence on its impact on care and outcomes. Further, drawing a small sample size of vapers from a population with a known, large incidence rate, such as ARDS, also raises the concern as to whether vaping status is being recorded in specific settings or subpopulations, such as on behavioral intake forms and/or psychiatric patients. If this is true, a selection bias may be present in the study.

Another limitation of this study was the inability to further stratify patients who vape based on the frequency and duration of vaping per day, the device manufacturer or model used, and the vape solution or contents used. The current method of recording vaping status is limited, as mentioned above. A better method for documentation of these details is needed to further identify, stratify, and examine their impact on outcomes of ARDS and other vaping-related conditions.

Literature examining the impact of vaping on ARDS hospital outcomes is lacking, thus making it difficult to compare and contrast our results with those. There is a similar article that examines ARDS outcomes; however, it focuses on smoking rather than vaping. Trieu et al. found no significant impact of smoking on ARDS outcomes, which is similar to our findings regarding vaping [13]. Literature continues to emerge highlighting vaping as a risk factor for ARDS; however, studies examining its impact on the outcomes in patients with ARDS are limited [14].

## Conclusions

The objective of this study was to examine the impact of vaping on hospital outcomes in patients with ARDS. While the study is limited by its sample size, it showed that vaping status had no statistical difference in mortality, hospital and ICU length of stay, need for NIPPV, and duration of mechanical ventilation among patients with ARDS. The only notable statistical difference was the need for mechanical ventilator support, with those who vaped having significantly decreased odds. Despite its limitations, this study shares our preliminary findings on the relationship between vaping and ARDS and highlights potential future research directions on the topic. Future studies can focus on implementing education or interventions to obtain more thorough vaping history, such as duration, frequency, model of vape, and contents of vape solution used to better gather vaping status data from at-risk populations. Also, future studies could utilize a national database to obtain a larger sample size and clarify the results presented here. Given the rising prevalence of vaping, a clearer understanding of its effects on pulmonary health and hospital outcomes remains a critical area for ongoing investigation.
